# Epigenome-wide association study for perceived discrimination among sub-Saharan African migrants in Europe - the RODAM study

**DOI:** 10.1038/s41598-020-61649-0

**Published:** 2020-03-18

**Authors:** Loes C. van der Laan, Karlijn A. C. Meeks, Felix P. Chilunga, Charles Agyemang, Andrea Venema, Marcel M. A. M. Mannens, Mohammad H. Zafarmand, Kerstin Klipstein-Grobusch, Liam Smeeth, Adebowale Adeyemo, Peter Henneman

**Affiliations:** 1 Department of Public Health, Amsterdam University Medical Centers, University of Amsterdam, Meibergdreef 9, 1105AZ Amsterdam, The Netherlands; 2 Center for Research on Genomics and Global Health, National Human Genome Research Institute, National Institutes of Health, 12 South Drive, MSC 5635, Bethesda, MD 20892-5635 United States; 3 Department of Clinical Genetics, Amsterdam University Medical Centers, University of Amsterdam, Meibergdreef 9, 1105AZ Amsterdam, The Netherlands; 4 Department of Clinical Epidemiology and Biostatistics, Amsterdam Public Health Research Institute, Amsterdam University Medical Centers, University of Amsterdam, Meibergdreef 9, 1105AZ Amsterdam, The Netherlands; 5 Julius Global Health, Julius Center for Health Sciences and Primary Care, University Medical Center Utrecht, Utrecht University, Heidelberglaan 100, 3584 CX Utrecht, The Netherlands; 6 Division of Epidemiology & Biostatistics, School of Public Health, Faculty of Health Sciences, University of the Witwatersrand, 1 Jan Smuts Avenue, Braamfontein, Johannesburg 2000 South Africa; 7 Department of Non-communicable Disease Epidemiology, London School of Hygiene and Tropical Medicine, Keppel St, Bloomsbury, London, WC1E 7HT UK

**Keywords:** Epigenetics, DNA methylation

## Abstract

Sub-Saharan African (SSA) migrants in Europe experience psychosocial stressors, such as perceived discrimination (PD). The effect of such a stressor on health could potentially be mediated via epigenetics. In this study we performed an epigenome-wide association study (EWAS) to assess the association between levels of PD with genome-wide DNA methylation profiles in SSA migrants. The Illumina 450 K DNA-methylation array was used on whole blood samples of 340 Ghanaian adults residing in three European cities from the cross-sectional Research on Obesity and Diabetes among African Migrants (RODAM) study. PD was assessed using sum scores of the Everyday Discrimination Scale (EDS). Differentially methylated positions and regions (DMPs and DMRs) were identified through linear regression analysis. Two hypo-methylated DMPs, namely cg13986138 (*CYFIP1*) and *cg10316525*(*ANKRD63*), were found to be associated with PD. DMR analysis identified 47 regions associated with the PD. To the best of our knowledge, this survey is the first EWAS for PD in first generation SSA migrants. We identified two DMPs associated with PD. Whether these associations underlie a consequence or causal effect within the scope of biological functionality needs additional research.

## Introduction

International migration from low- and middle income to high income countries has been increasing over the years and migrants face a plethora of psychosocial stressors in the host countries, including perceived discrimination (PD)^1^. In 2014, more than half (55%) of all migrants living in the Netherlands perceived some form of discrimination^2^. Ethnic background, religious beliefs, and skin colour play an important role in PD among migrants. However, the nature of discrimination varies with context^2^. For example, sub-Saharan African (SSA) migrants, one of the fastest growing groups of migrants in Europe today, have reported discrimination across a broad range of domains, such as public spaces, nightlife, public institutions (police, schools, etc.), workplace, terms of employment, job seeking, education and access to healthcare. PD has been associated with socio-economic consequences and deleterious effects on health^2,3^.

Several studies have shown that PD is associated with non-communicable diseases such as depression, hypertension, type 2 diabetes, obesity and cardiovascular diseases^3–9^. Our previous work from the Research on Obesity and Diabetes among African Migrants (RODAM) study, showed that these cardio-metabolic outcomes disproportionally affect SSA migrants in Europe. To date, the underlying causes of this disproportionate burden among SSA migrants and the biological mechanisms underlying these associations remain unclear^4,9–11^.

Epigenetics is the study of heritable yet reversible molecular modifications to the DNA without altering the DNA sequence, caused by environmental triggers^12,13^. Epigenetic mechanisms include histone modifications, ncRNAs and DNA methylation of which the latter is the most commonly studied in epigenetics. DNA methylation involves the binding of methyl groups to CpG sites on the DNA. Epigenetics, and thus DNA methylation, can be affected by environmental factors^14^. Psychosocial stress has been linked to changes in DNA methylation of genes implicated in the stress response system^15^. These genes include the glucocorticoid receptor gene (*NR3C1*), serotonin transporter gene (*SLC6A4*) and the corticotrophin-releasing factor (*CRF*) gene^15–17^. Since epigenetics is known to be affected by environmental factors, we hypothesized that exposure to discrimination might result in an altered state of DNA methylation, globally or at specific loci^13^. Previous reported studies showed that PD represents an important factor associated with the stress response system and mental health disorders and therefore represents relevant psychosocial stressor^18^.

To date, no study has assessed the association between discrimination and epigenetics in SSA migrants. In the present study, we conducted an epigenome-wide association study (EWAS) where we aimed to detect novel epigenetic loci associated with PD as assessed by the Everyday Discrimination Scale (EDS) in a cohort of Ghanaian migrants from the RODAM study.

## Results

We included 340 Ghanaian migrants from the Research on Obesity and Diabetes among African Migrants (RODAM) study. Almost one third (30.8%) of migrants reported work as reason for migration (Table 1). The mean age was about 50 years. Men were slightly overrepresented in the sample (54%), and sex showed a low correlation (rho = −0.14) with the summed EDS. 42% reported secondary education as highest level of education obtained, 60.3% received income from wages, own company or investments. Unskilled manual labour and lower non-manual labour were the most commonly reported occupations. On average, migrants resided 19 years in Europe and felt more Ghanaian than English/German/Dutch. The median total EDS score for the 340 migrants was 16 (IQR, 9,21) out of a possible EDS score ranging from 9 (never on all items) to 45 (very often on all items) (Table 1). Feeling English/German/Dutch was significantly correlated with the summed EDS (rho = −0.116), whereas feeling Ghanaian and length of stay in Europe were not. We evaluated 3 indicators for socio-economic status (SES) and found source of income correlated with the summed EDS (rho = −0132), but not attained education level nor occupation. Furthermore, we observed no correlation between smoking or estimated distribution of CD8+ T cells, CD4+ T cells, Natural Killer Cells, B cells and Granulocytes and summed EDS, but we did observe a low correlation between the estimated relative distribution of Monocytes (spearman’s rho of 0.12).

**Table 1:** Descriptive statistics of participants included in analyses.

		All	Spearman’s rho with sum EDS
N, (%)		340 (100%)	
**Demographics**			
Age, years^#^		49.8 (48.7; 50.8)	0.00
Sex, female, (%)		155 (45.6%)	−0.14*
**PD and migration-related factors**			
EDS, (25^th^,75^th^ percentile)^##^	Total	16 (9, 21)	
	Female	14 (9, 20)	
	Male	17 (11, 22)	
Length of stay in Europe, years^#^		19.2 (18.2; 20.2)	0.073
Work as reason for migration		104 (30.6%)	0.068
Feeling English/German/Dutch	Totally agree	19 (5.6%)	−0.116*
	Agree	72 (21.2%)	
	Neutral	90 (26.5%)	
	Disagree	74 (21.8%)	
	Totally disagree	71 (20.9%)	
Feeling Ghanaian	Totally agree	187 (55%)	0.054
	Agree	107 (31.5%)	
	Neutral	31 (9.1%)	
	Disagree	4 (1.2%)	
	Totally disagree	6 (1.8%)	
**Socio-economic status (SES) indicators**			
Education level, (%)	Never been to school/elementary only	72 (21.6%)	0.07
	Lower vocational or lower secondary schooling	139 (41.6%)	
	Intermediate vocational or intermediate/higher secondary schooling	73 (21.9%)	
	Higher vocational schooling or university	50 (15.0%)	
Source of income	Wages or income from own company or investments	205 (60.3%)	−0.132*
	Pension	16 (4.7%)	
	Some form of benefits	44 (12.9%)	
	Student loan	3 (0.9%)	
	Combined wages + benefits	29 (8.5%)	
	Other e.g. remittance	13 (3.8%)	
	Missing	30 (8.8%)	
Occupation, (%)	Upper non-manual	40 (11.8%)	−0.008
	Lower non-manual	68 (20.0%)	
	Skilled manual	21 (6.2%)	
	Unskilled manual	129 (37.9%)	
	Farmer	2 (0.6%)	
	Missing	80 (23.5%)	
**Factors with the potential to affect DNA methylation**			
Smoking, yes, (%)		13 (3.8%)	0.04
Estimated cell counts, % (IQ)^#^	CD8+ cells	11.0 (10.5, 11.4)	0.03
	CD4+ cells	18.0 (17.4, 18.5)	0.04
	NK cells	9.2 (8.6, 9.7)	−0.05
	B cells	9.7 (9.4, 10.1)	0.12*
	Monocytes	8.3 (8.1, 8.6)	−0.05
	Granulocytes	47.4 (46.4, 48.3)	−0.05

Numbers are in n (%) for categorical variables. ^#^Mean (95%CI) for normally distributed continuous variables. ^##^Median (25th percentile, 75th percentile) for non-normally distributed variables. *Spearman’s correlation coefficient (rho) at a p < 0.05, **Spearman’s correlation coefficient (rho) at a p < 0.01.

**Figure 1:**
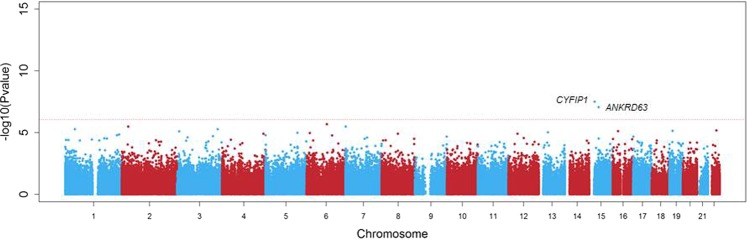
Manhattan plot of differentially methylated positions for the continuous model of PD in Ghanaian migrants in Europe. The DMPs, annotated to the *CYFIP1* gene and the *ANKRD63* gene, are a significantly differently methylated position for perceived discrimination. *The red line represents genome-wide significance, FDR* < *0.05*.

**Figure 2:**
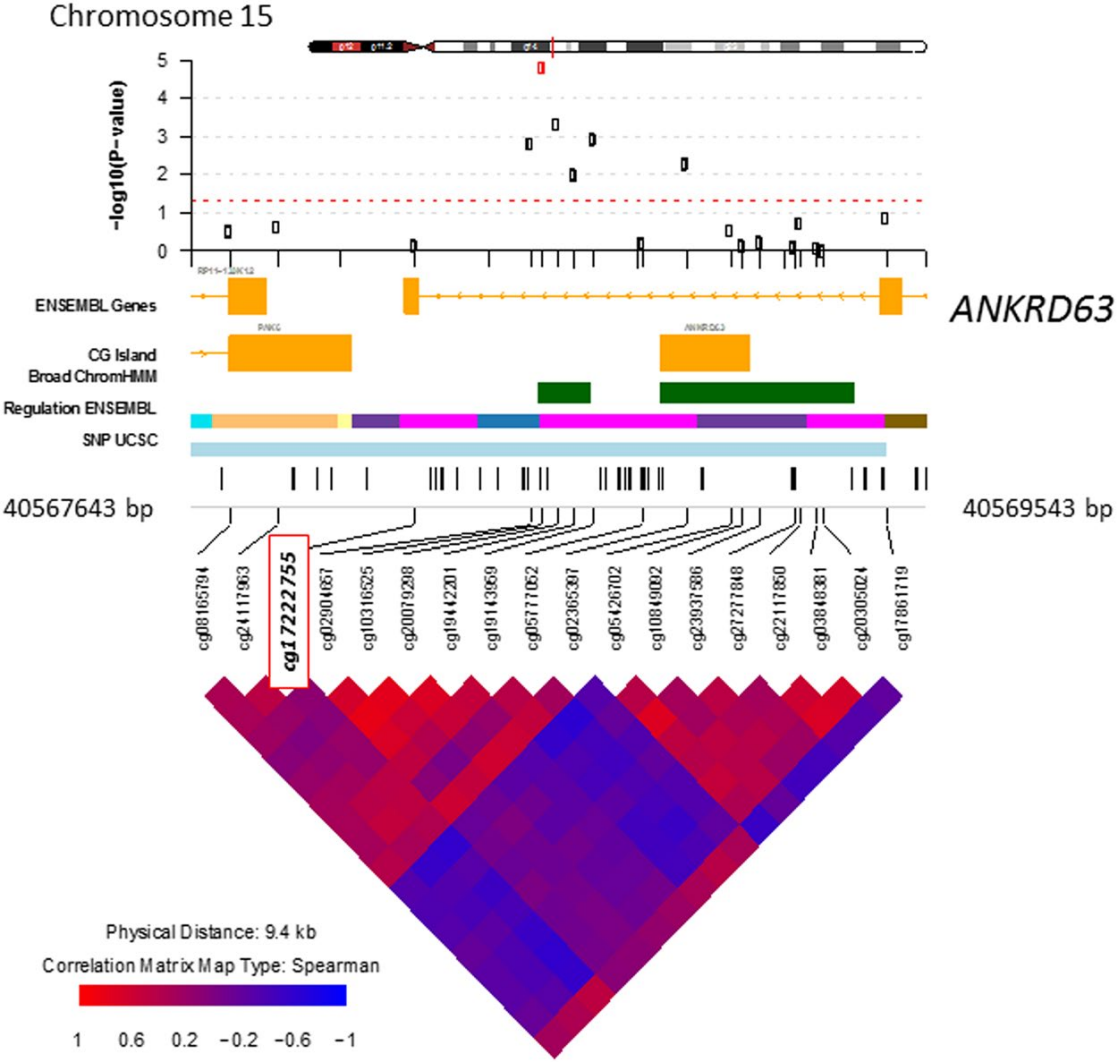
Differentially Methylated Position (DMP) annotated to gene *CYFIP1* associated with PD. The DMP is annotated as the CpG site in the red square with a 5 kb region (hg19) visualized around the DMP. Broad ChromHMM visualizes the omic feature of the CpG site; bright blue, weak Txn; orange, weak enhancer; yellow, weak enhancer; purple, poised promoter; pink, repressed; blue, insulator; brown, heterochromatin.

**Figure 3:**
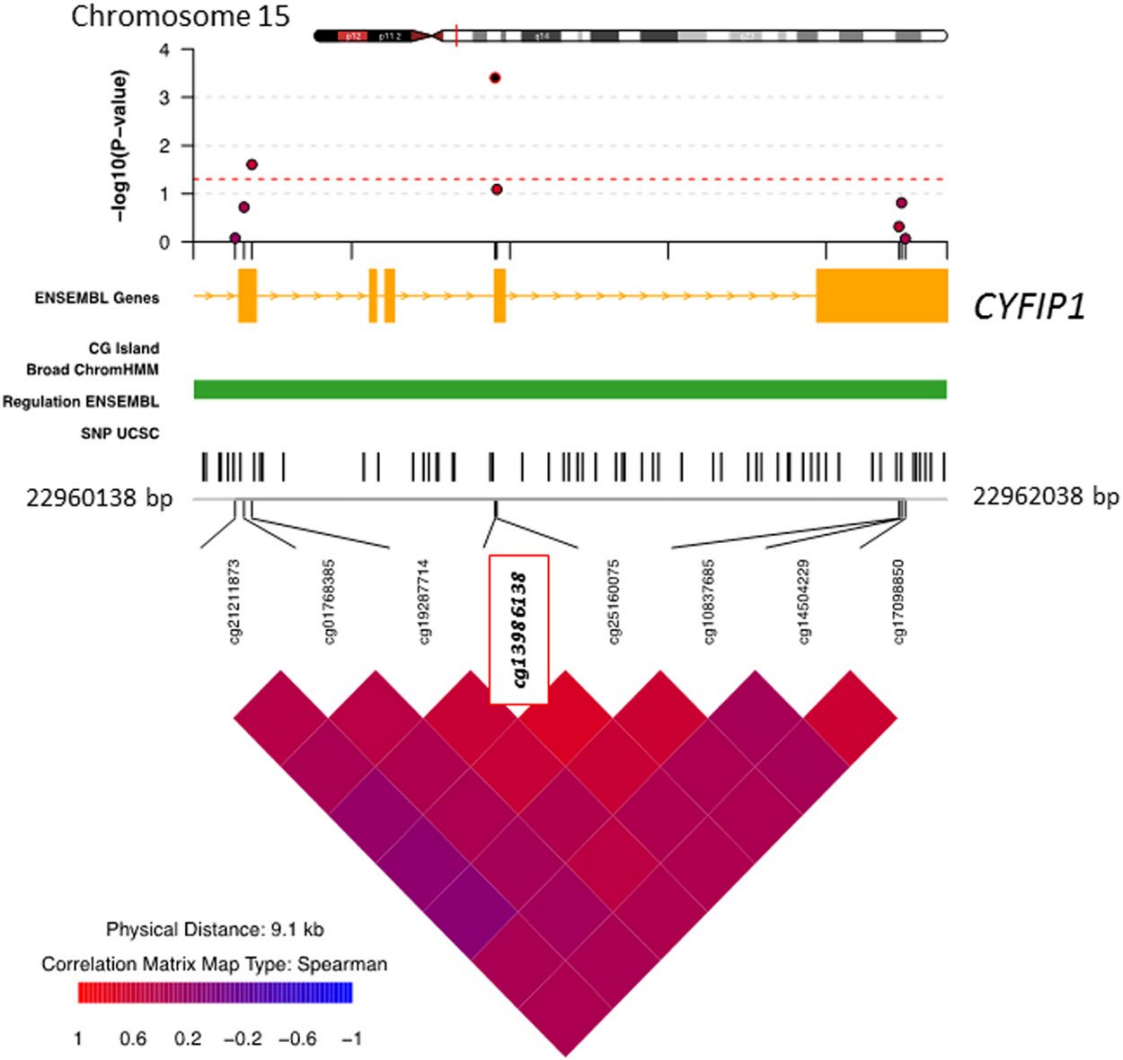
Differentially Methylated Position (DMP) annotated to gene *ANKRD63* associated with PD. The DMP is annotated as the CpG site in the red square with a 5 kb region (hg19) visualized around the DMP. Broad ChromHMM visualizes the omic feature of the CpG site; bright blue, weak Txn; orange, weak enhancer; yellow, weak enhancer; purple, poised promoter; pink, repressed; blue, insulator; brown, heterochromatin.

Next, we performed differentially methylation analysis using linear regression analyses on the EDS as a continuous measure for PD. We observed two differential methylated positions (DMPs), cg13986138 (FDR = 0.014) and cg10316525 (FDR = 0.019), that were epigenome-wide significant associated with PD (Table 2 and Fig. 1). The DMP (cg13986138) that was annotated to gene body of the *CYFIP1* gene, (also known as Specifically Rac1-associated Protein1;SRA1), was found to be 0.072% hypo methylated per 1 point increase in the EDS sum score (Table 2). The DMP annotated to intergenic region of the *ANKRD63* gene (cg10316525) was 0.144% hypo methylated per 1 point increase in the EDS sum score. Figures 2 and 3 visualize the flanking regions around the DMPs annotated to the *CYFIP1 and ANKRD63* respectively. We did not observe any known active regulatory elements at or nearby cg13986138 (*CYFIP1*). However, we were able to tag several known active regulatory elements around cg10316525 (*ANKRD63)*, e.g. poised promoters and insulators.

**Table 2:** Top 5 Differentially Methylated Positions associated with PD (continuous trait summed EDS association).

CpG	Chr	Position	Gene	Feature^c^	Delta β^d^	p-value	FDR	p-value BACON	FDR BACON
cg13986138	15	22963760	*CYFIP1*	Body	−7.49E^−4^	7.61E^−8^	0.016	3.27E^−08^	0.014
cg10316525	15	40572137	*ANKRD63*^a^	IGR	−1.46E^−3^	9.81E^−9^	0.042	8.93E^−08^	0.019
cg12337011	2	28002424	MRPL33	3′UTR	−1.24E^−3^	2.21E^−5^	0.800	3.32E^−06^	0.288
cg13145550	7	1350879	UNCX^b^	IGR	−6.57E^−3^	2.64E^−5^	0.800	3.36E^−06^	0.288
cg15457795	3	9747183	CPNE9	Body	−8.86E^−4^	1.78E^−5^	0.800	8.15E^−06^	0.318

^a^CpG is located approximately 15 kb downstream of *ANKRD63*. ^b^CpG is located approximately 80 kb downstream of *UNCX*. ^c^Based on manifest feature annotation Illumina (hg19). ^d^Negative beta-values indicate lower DNA methylation (hypo-methylation) in cases compared to controls. FDR < 0.05 was considered significant.

Subsequently we performed differentially methylated region (DMR) analysis. We detected in total 47 DMRs that showed a family wise error rate (FWER) < 0.2 and that included more than two thirds of the probes within the cluster (Supplementary Table 1). The following genome-wide significant DMRs were detected, all annotated to intergenic regions: (1) one DMR located at chromosome 17 approximately 1 kb downstream of *ALOX15P1* (FWER = 0.006), (2) a second DMR located at chromosome 15 in the hCG_2003567 gene (FWER = 0.04) and finally (3) a third DMR located at chromosome 2 approximately 2 kb downstream of the STF2D3 gene (FWER = 0.048).

Finally, the 47 observed DMRs were submitted for gene set enrichment analysis. Three enriched GO categories were found for continuous analysis; three cellular component pathways of which two are involved in cell projection and one in neuron projection (Table 3). None of the cellular component pathway contained a genome-wide significant DMP.

**Table 3:** Gene set Enrichment analysis, significant pathways including DMPs associated with PD.

Pathway name	Pathway ID	Database	Genes in pathway	C	O	E	R	P-value	FDR
Cell projection part	GO:0044463	Cellular component	*PTPRN2, KCNQ2, RGS14, PALM, CATSPER4, FZD7*	639	6	0.94	6.40	3.0E^−04^	0.012
Neuron projection	GO:0043005	Cellular component	*PTPRN2, KCNQ2, RGS14, PALM, FZD7*	651	5	0.95	5.24	2.2E^−03^	0.029
Cell projection	GO:0042995	Cellular component	*PTPRN2, ROPN1B, KCNQ2, RGS14, PALM, CATSPER4, FZD7*	1230	7	1.80	3.88	1.5E^−03^	0.029

DMP is highlighted in bold, C: the number of reference genes in the category, O: the number of genes in the gene set and the category, E: the expected number in the category, R: ratio of enrichment, p-value: p value from hypergeometric test, FDR: p value adjusted by the FDR multiple test adjustment. FDR < 0.05 was considered significant.

## Discussion

This study describes the first EWAS for perceived discrimination in first generation SSA migrants in Europe. A major strength of this research is the relatively homogenous SSA study population, i.e. all Ghanaian, which is necessary to study the environmental influences on the epigenome with limited potential bias caused by population stratification.

We detected two novel DNA methylation loci, cg13986138 (*CYFIP1*) and cg10316525 (*ANKRD63*), that were associated with PD in Ghanaian migrants in Europe. The DMP annotated to *CYFIP1*, also known as Specifically Rac1-associated Protein1 (SRA1) or p140SRA-1, showed an inverse relationship between methylation and PD, i.e. was found hypo-methylated for increasing scores of perceived discrimination. *CYFIP1* is a protein coding gene that is involved in two molecular processes: the FMRP-elF4E translation complex and the Wave Regulatory Complex (WRC)^19,20^. These complexes are essential for synaptic morphology and function regulated by inhibition of local protein synthesis and favouring actin remodelling^19^. Notably,*CYFIP1* mediates the translational repression activity of Fragile X Mental Retardation Protein (FMRP) in the brain in the Fragile X Syndrome (FXS)^21^. In addition, since *CYFIP1* provides the binding site for the complex to Rac1 this gene might also have a role in cancer (GTEx Portal) (www.gtexportal.org) and type 2 diabetes mellitus^22^. Cg13986138 is annotated to the gene body of the *CYFIP1* gene and expressed in multiple tissues including brain tissue, lung tissue and blood (GTEx Portal) (www.gtexportal.org). In general, hypo-methylation in the gene-body results in a reduction of gene expression while hypo-methylation in the promoter region of a gene is often associated with increased expression^23^. Therefore, hypo-methylation of the DMP annotated to the *CYFIP1* gene (−0.072%) may indicate a lower gene expression in individuals experiencing higher levels of perceived discrimination. Hypo-methylation of the DMP annotated *CYFIP1* gene could lead to dysregulation of synaptic function and morphology. However, as differences in DNA methylation are low, differences in *CYFIP1* gene expression are presumably of limiting impact. This low variation in DNA methylation of this locus could indicate that the heterogeneity of the tissue, i.e. whole blood, diminishes the effect size, although estimates of these different types of cells were included in the model. Further translational research, targeting specific brain cells, could give insight into whether the activity of locus is truly associated with its epigenetic state and whether this locus is indeed susceptible for environmental exposure.

The DMP annotated to *ANKRD63* showed an inverse relationship between methylation and PD and was annotated to an intergenic region. Intergenic regions can involve several possible functions, i.e. enhancer regions or intergenic transcription factor binding sites^24^. The DMP annotated gene has been previously found to be expressed in brain tissue, more specifically the Nucleus Accumbens, Putamen and the Caudate (GTEx Portal) (www.gtexportal.org). *ANKRD63* is a protein-coding gene that is part of the Ankyrin (ANK) repeat proteins. In general, ANK-repeat proteins are involved in various physiological processes in which they function exclusively through mediating specific protein-protein interactions^25^. As the function of the intergenic region is not yet fully determined and can differ, the DNA may or may not encode regulatory functions^26,27^. However, to date, little is known about the exact function of the *ANKRD63* gene. Consequently, it is difficult to speculate on the effect that hypo-methylation (−0,144%) in the intergenic region of the *ANKRD63* gene may have. Previous research has indicated that the *ANKRD63* protein is associated with mental health disorders^28^. Recently, de Mendoza *et al*. (2018) reported nine DMPs that were associated with PD in the African-American population^29^. In this study, it was shown that nine DMPs were genome-wide significant associated with Major Life Discrimination. Although the design and sample of the study of Mendoza *et al*. differ in great detail from ours, we performed a replication study on these nine reported DMPs. None of the nine DMPs reported by Mendoza *et al*. were present in our top1000 DMPs (Supplementary Table 1). The fact that our study represents probably less genetic admixture, included male and female participants, and included a different measure of PD compared to the study of Mendoza *et al*., might explain the absence of replication. In order to detect more general aberrant methylated loci that are associated with PD, a larger sample size is needed, e.g. in combining multiple PD EWAS in meta-analysis approaches.

The present study shows that PD is associated with aberrant DNA methylation of two specific regions. While this study provides clues for further study of the biological mechanisms underlying a possible association between discrimination and epigenetics, further research is needed to elucidate whether the epigenetic aberrations we have detected are the consequence or cause of perceived discrimination. Studies in similar migrant populations are essential to validate and refine this association in order to improve migrant well-being. Studies assessing the association between discrimination and the epigenome could complement the EDS score with a more objective quantitative measure, e.g. cortisol levels or Magnetic Resonance Imaging (MRI) data. The use of an objective measure of stress, such as cortisol, will certainly add validity and objectivity to the use of the self-reported EDS.

This study is subjected to several limitations. The use of the self-reported EDS, which is susceptible to recall bias, potentially increases the phenotypic heterogeneity within the cohort and thereby limiting our statistical power. However, the EDS has demonstrated good internal consistency, stability over time and convergent and divergent validity in previous research indicating that the EDS was a good measure for PD^30–34^. DNA methylation was assessed in whole blood samples. Given the tissue specific nature of DNA methylation is tissue specific, ideally this study would have analysed target tissue in which the *CYFIP1* and *ANKRD63* genes are expressed, including brain tissue. However, the availability of these tissues for epidemiological studies is limited. Nevertheless, research indicates that the use peripheral blood samples provides a valuable impression and is relatively consistent with other tissue types^35^. In this context we submitted the two genome-wide significant probes to the online “Blood Brain DNA Methylation comparison Tool” (www.epigenetics.iop.kcl.ac.uk/bloodbrain)^36^. This evaluation showed a positive but limited correlation of the DNA-methylation of cg13986138 (*CYFIP*) between blood and brain tissues (cerebellum, r = 0.17, P = 0.148) and positive moderate correlations between the DNA-methylation of cg10316525 (*ANKRD63*) between blood and brain tissues (entorhinal cortex, r = 0.29, P = 0.014; superior temporal gyrus, r = 0.28, P = 0.014), suggesting that our results detected in whole blood indeed might reflect at least in part the epigenome in specific brain tissue. We were unable to stratify the analyses by sex due to the limited sample size. There could be a difference in the association between PD and DNA metylation between men and women. We, therefore, compared mean percentage methylation of the novel DMPs identified between men and women. For the novel DMPs identified, we found that cg13986138 mean methylation level did not differ between men (mean = 90.8%) and women (mean = 90.9%) (p-value = 0.77), while cg10316525 was significantly higher methylated in women (mean = 95.3%) than in men (93.8%) (p-value < 0.001). Future studies should consider sex-specific associations between discrimination and DNA methylation. Lastly, the cross-sectional design of the RODAM study precludes causal interpretations.

In conclusion, this study was the first EWAS in first generation SSA migrants assessing the association between PD and DNA methylation. We identified two epigenetic loci that were associated with perceived discrimination. Although further research is essential to validate the results, these findings provide a basis to further investigate the biological mechanisms underlying adverse health consequences of discrimination in migrants.

## Methods

This study was based on data from the RODAM study. Ethical approval, in accordance with the ethical standards as laid down in the 1964 Declaration of Helsinki and its later amendments or comparable ethical standards, was obtained before the start of data collection from the ethics committees of the following involved institutions: *Ghana*, School of Medical Sciences/Komfo Anokye Teaching Hospital Committee on Human Research, Publication & Ethical Review Board; *The Netherlands*, Institutional Review Board of the AMC, University of Amsterdam; *Germany*, Ethics Committee of Charite-Universitätsmedizin Berlin and the *United Kingdom*, London School of Hygiene and Tropical Medicine Research Ethics Committee. Informed written consent was obtained from all participants prior to the enrolment in the RODAM study.

The RODAM study aims to unravel the underlying factors for the high prevalence of type 2 diabetes mellitus and obesity among SSA migrants with a focus on the interaction between environmental exposures and genetics. Details on the study design and data collection have previously been described elsewhere^10^. In brief, between 2012 and 2015 Ghanaian adults (19–96 years of age) were recruited in rural Ghana, urban Ghana, Amsterdam, London and Berlin. In the present analysis only Ghanaian migrants in Europe were included. Ghanaian origin was defined as being born in Ghana and having at least one parent born in Ghana or not born in Ghana but having both parents born in Ghana. In Amsterdam, Ghanaian participants were randomly drawn from the Amsterdam Municipal Register, which holds data on country of birth of citizens and their parents. In London, Ghanaian organizations served as the sampling frame as there was no population register for migrant groups. In Berlin, member lists of Ghanaian churches and organizations served as the sampling frame.

This EWAS used a subset of the RODAM data for which epigenetics data are available (n = 744). After exclusions based on quality control criteria and missing phenotypic data (n = 81), 663 participants remained for analyses. To avoid bias due to conceptual variation in PD between Ghana and Europe, only migrant Ghanaians in Europe were included for epigenetic analysis. In total 340 Ghanaian migrants were included, of whom 143 resided in Amsterdam, 80 in Berlin and 117 in London.

Information on demographics and self-reported levels of PD were collected by a self- or interviewer-administered questionnaire. PD was assessed with the Everyday Discrimination Scale (EDS)^34^. Based on the construct defined by Essed^37^ the EDS attempts to measure everyday perceived discrimination, which is discrimination as a structured and chronic part of relatively minor experiences of unfair treatment. The EDS has demonstrated good internal consistency, stability over time, and convergent and divergent validity in prior studies^30–34^. Within this subset of the RODAM dataset, the EDS displayed a Cronbach’s Alfa of 0.929, indicating excellent internal consistency.

The EDS included nine questions that represent nine events. Participants were asked to indicate how often these nine events happened to them in their daily life. The EDS was scored on a five-point Likert scale; 1 - “Never”, 2 - “Hardly ever”, 3 - “Not too often”, 4 - “Fairly often”, 5 - “Very often”. The sum of these scores resulted in a minimum overall score of 9 (scoring “Never” for each item) to a maximum overall score of 45 (scoring “Very often” for each item). Hence, a higher score indicated more perceived discrimination. Items were considered missing if more than one item was missing (n = 53). If only one item was missing the mean score of the other items (rounded to whole numbers) was used to impute the missing item (n = 15). Participant characteristics on age, sex, smoking behaviour, education, estimated blood cell types (described in detail below) and their relation with PD (summed EDS) were evaluated according spearman’s correlation using SPSS (v20, IBM).

Assessment of the epigenetic profiles, its processing, and quality control within in the RODAM study were described previously^38^. In brief, fasting whole blood samples were shipped to the Source Bioscience Nottingham, UK, where DNA extraction and DNA methylation profiling was performed. Genome-wide DNA methylation profiles were obtained using the Illumina Human Methylation 450 K beadchip. This array measures approximately 485,000 CpG sites. Quality control was performed using the *MethylAid* software(version 1.4.0.). Probes annotated to the X, and Y chromosomes, known to involve cross hybridization or to involve a (common) SNP were removed from the dataset, resulting in a total set of 429,419 CpG sites. Blood cell mixture estimation was based on the method described by Houseman *et al*.^39^.

We performed association analyses to detect differentially methylated positions (DMPs) and differentially methylated regions (DMRs) for PD (summed EDS). For detection of DMPs, linear regression (lmfit) analyses were performed for the continuous PD variable in “R” with the *minfi* and *limma* package using DNA methylation levels as dependent variable. Age, sex, blood cell distribution and technical effects (hybridization batch and plate position) were included as covariates. Within our sample of 340 participants, the first eight principal components of the genome-wide methylation data were subjected to correlation analyses with potential covariates in order to detect additional confounders or bias (Supplementary File 1 – Supplementary Figs. 1 and 2). False Discovery Rate (FDR) adjustment was used to adjust for multiple testing, assuming a FDR of <0.05 statistically significant. The *BACON* package (version 1.4.0) in “R” software was applied to address possible inflation of our test statistics by systematic biases^40^. The Bayesian method of this package proposes a bias and inflation correction based on the construction of an empirical null distribution. Q-Q plots were used for model fitting evaluation (Supplementary File 1 – Supplementary Fig. 3). The inflation measure lambda increased from 0.79 to 1.02, after implementation of the *BACON* package.

The *bumphunter* function in the *minfi* package was used to identify DMRs. Similar to the DMP analyses, we fitted models for the EDS as continuous measure including age, sex, estimated cell type distributions and technical effects as covariates. Default settings were used with exception of bootstrapping, for which we applied 500 permutations. The number of DMRs was optimized using a cut-off of 0.075, corresponding to a 7.5% difference in beta values. Methylation cut-offs were optimized based on effect sizes and significance levels in volcano plot of the DMP analysis. DMRs were defined as three or more CpG sites within the cluster. A Family Wise Error Rate (FWER) of <0.05 was assumed statistically significant.

In order to evaluate our findings in a broader and biological context, pathway analyses were performed using the “WEB-based Gene set AnaLysis Toolkit” (WebGestalt) (www.webgestalt.org). This gene set analysis toolkit was used to determine Biological Process, Cellular Component and Molecular Function pathways according the Gene Ontology (GO) terms. We submitted all DMRs with a FWER < 0.200 for continuous (n = 47) in GSE analyses using the following settings: hsapiens, hsapiens_gene_symbol, GO analysis, hsapiens_genome, hypergeometric, BH, significance level q < 0.05, minimum number of genes for category = 4.

## Supplementary information

Supplementary File 1.

Supplementary Table 1.

## Data Availability

The datasets used and/or analysed during the current study are available from the corresponding author on reasonable request and data transfer agreement.

## References

[1] OECD. *International Migration Outlook 2019*, https://www.oecd-ilibrary.org/content/publication/c3e35eec-en (2019).

[2] Andriessen, I., Fernee, H. & Wittebrood, K. Perceived discrimination in the Netherlands, https://www.scp.nl/english/Publications/Publications_by_year/Publications_2014/Perceived_discrimination_in_the_Netherlands (2014).

[3] Chilunga FP (2019). Perceived discrimination and stressful life events are associated with cardiovascular risk score in migrant and non-migrant populations: The RODAM study. Int. J. cardiology.

[4] Williams DR, Mohammed SA (2009). Discrimination and racial disparities in health: evidence and needed research. J. Behav. Med..

[5] Williams DR, Neighbors HW, Jackson JS (2003). Racial/ethnic discrimination and health: findings from community studies. Am. J. public. health.

[6] Cozier, Y. C. *et al*. Racism, segregation, and risk of obesity in the Black Women’s Health Study. *American journal of epidemiology*, kwu004 (2014). 10.1093/aje/kwu004 PMC3969538 24585257

[7] Pascoe EA, Smart Richman L (2009). Perceived discrimination and health: a meta-analytic review. Psychological Bull..

[8] Ikram UZ (2017). Perceived Ethnic Discrimination and the Metabolic Syndrome in Ethnic Minority Groups: The Healthy Life in an Urban Setting Study. Psychosom. Med..

[9] Agyemang C (2016). Obesity and type 2 diabetes in sub-Saharan Africans–Is the burden in today’s Africa similar to African migrants in Europe? The RODAM study. BMC Med..

[10] Agyemang C (2014). Rationale and cross-sectional study design of the Research on Obesity and type 2 Diabetes among African Migrants: the RODAM study. BMJ open..

[11] Boateng D (2016). Migration and cardiovascular disease risk among Ghanaian populations in Europe: The RODAM study. Eur. J. Public. Health.

[12] Egger G, Liang G, Aparicio A, Jones PA (2004). Epigenetics in human disease and prospects for epigenetic therapy. Nat..

[13] Meaney MJ (2010). Epigenetics and the biological definition of gene× environment interactions. Child. Dev..

[14] Alegría-Torres, J. A., Baccarelli, A. & Bollati, V. Epigenetics and lifestyle. (2011). 10.2217/epi.11.22 PMC3752894 22122337

[15] Unternaehrer, E. & Meinlschmidt, G. In *Epigenetics and Neuroendocrinology* 227–261 (Springer, 2016).

[16] Gudsnuk K, Champagne FA (2012). Epigenetic influence of stress and the social environment. ILAR J..

[17] Kuzawa CW, Sweet E (2009). Epigenetics and the embodiment of race: developmental origins of US racial disparities in cardiovascular health. Am. J. Hum. Biol..

[18] Mama SK (2016). Psychosocial mechanisms linking the social environment to mental health in African Americans. PLoS one.

[19] De Rubeis S (2013). CYFIP1 coordinates mRNA translation and cytoskeleton remodeling to ensure proper dendritic spine formation. Neuron.

[20] Kobayashi K (1998). p140Sra-1 (specifically Rac1-associated protein) is a novel specific target for Rac1 small GTPase. J. Biol. Chem..

[21] Napoli I (2008). The fragile X syndrome protein represses activity-dependent translation through CYFIP1, a new 4E-BP. Cell.

[22] Koronakis V (2011). WAVE regulatory complex activation by cooperating GTPases Arf and Rac1. Proc. Natl Acad. Sci. USA.

[23] Jones PA (2012). Functions of DNA methylation: islands, start sites, gene bodies and beyond. Nat. Rev. Genet..

[24] Hindorff LA (2009). Potential etiologic and functional implications of genome-wide association loci for human diseases and traits. Proc. Natl Acad. Sci. USA.

[25] Sedgwick SG, Smerdon SJ (1999). The ankyrin repeat: a diversity of interactions on a common structural framework. Trends biochemical Sci..

[26] Niu D-K, Jiang L (2013). Can ENCODE tell us how much junk DNA we carry in our genome?. Biochemical biophysical Res. Commun..

[27] Qu H, Fang X (2013). A brief review on the Human Encyclopedia of DNA Elements (ENCODE) project. Genomics, Proteom. Bioinforma..

[28] Consortium”, S. W. G. O. T. P. G (2014). Biological insights from 108 schizophrenia-associated genetic loci. Nat..

[29] Kilpatrick Quentin K., Taylor John (2018). Racial/Ethnic Contrasts in the Relationships between Physical Disability, Perceived Discrimination, and Depressive Symptoms. Journal of Racial and Ethnic Health Disparities.

[30] Taylor TR, Kamarck TW, Shiffman S (2004). Validation of the Detroit Area Study Discrimination Scale in a community sample of older African American adults: the Pittsburgh healthy heart project. Int. J. Behav. Med..

[31] Krieger N, Smith K, Naishadham D, Hartman C, Barbeau EM (2005). Experiences of discrimination: validity and reliability of a self-report measure for population health research on racism and health. Soc. Sci. Med..

[32] Kessler, R. C., Mickelson, K. D. & Williams, D. R. The prevalence, distribution, and mental health correlates of perceived discrimination in the United States. *Journal of health and social behavior*, 208–230 (1999). 10513145

[33] Lewis TT (2006). Chronic exposure to everyday discrimination and coronary artery calcification in African-American women: the SWAN Heart Study. Psychosom. Med..

[34] Williams DR, Yu Y, Jackson JS, Anderson NB (1997). Racial differences in physical and mental health: Socio-economic status, stress and discrimination. J. health Psychol..

[35] Kato N (2015). Trans-ancestry genome-wide association study identifies 12 genetic loci influencing blood pressure and implicates a role for DNA methylation. Nat. Genet..

[36] Hannon E, Lunnon K, Schalkwyk L, Mill J (2015). Interindividual methylomic variation across blood, cortex, and cerebellum: implications for epigenetic studies of neurological and neuropsychiatric phenotypes. Epigenetics.

[37] Essed, P. *Understanding everyday racism: An interdisciplinary theory*. Vol. 2 (Sage, 1991).

[38] Meeks KAC (2017). An epigenome-wide association study in whole blood of measures of adiposity among Ghanaians: the RODAM study. Clin. Epigenetics.

[39] Houseman EA (2012). DNA methylation arrays as surrogate measures of cell mixture distribution. BMC Bioinforma..

[40] van Iterson M, van Zwet EW, Heijmans BT (2017). Controlling bias and inflation in epigenome-and transcriptome-wide association studies using the empirical null distribution. Genome Biol..

