# Supplementary material for: Epigenome-wide association study for perceived discrimination among sub-Saharan African migrants in Europe - the RODAM study
Source: Sci Rep. 2020 Mar 18;10:4919. doi: 10.1038/s41598-020-61649-0 (PMC7080832; doi:10.1038/s41598-020-61649-0)
Supplement: Supplementary file 1 [file 41598_2020_61649_MOESM1_ESM.docx]

**Epigenome-wide association study for perceived discrimination among sub-Saharan African migrants in Europe - the RODAM study**

Loes C. van der Laan (MSc)^1≠^, Karlijn A.C. Meeks (PhD)^1≠^, Felix P. Chilunga (MD)^1^, Charles Agyemang (PhD)^1^, Andrea Venema (BSc)^2^, Marcel M.A.M. Mannens (PhD)^2^, Mohammad H. Zafarmand (PhD)^3^, Kerstin Klipstein-Grobusch (PhD)^4,5^, Liam Smeeth (MD, PhD)^6^, Adebowale Adeyemo (PhD)^7^, Peter Henneman (PhD)^2*^.

^1^Department of Public Health, Amsterdam University Medical Center, University of Amsterdam, , Meibergdreef 9, 1105AZ Amsterdam, The Netherlands. ^2^Department of Clinical Genetics, Amsterdam University Medical Center, University of Amsterdam, Meibergdreef 9, 1105AZ Amsterdam, The Netherlands. ^3^Department of Clinical Epidemiology and Biostatistics, Amsterdam Public Health Research Institute, Amsterdam University Medical Center, University of Amsterdam, Meibergdreef 9, 1105AZ Amsterdam, The Netherlands. ^4^Julius Global Health, Julius Center for Health Sciences and Primary Care, University Medical Center Utrecht, Utrecht University, Heidelberglaan 100, 3584 CX Utrecht, The Netherlands. ^5^Division of Epidemiology & Biostatistics, School of Public Health, Faculty of Health Sciences, University of the Witwatersrand, 1 Jan Smuts Avenue, Braamfontein, Johannesburg, 2000, South Africa. ^6^Department of Non-communicable Disease Epidemiology, London School of Hygiene and Tropical Medicine, Keppel St, Bloomsbury, London WC1E 7HT, UK. ^7^Center for Research on Genomics and Global Health, National Human Genome Research Institute, National Institutes of Health, 12 South Drive, MSC 5635, Bethesda, MD 20892-5635, United States.

≠Authors contributed equally to this work

**Supplementary file 1**

**Principle component analysis (PCA)**

A principle component analysis was performed to assess covariate inclusion. Gender, age, PD, hybridization batch, array position and estimated cell type distributions were included in principle component analysis. Correlation of estimated cell type distributions with DNA methylation is displayed in figure 1. Blood cell distributions were estimated using the method proposed by Houseman *et al* ^1^ and estimated cell type distributions were included as covariates in the analyses. Correlation of the covariates with DNA methylation principal component 1 to 8 is shown in figure 2. Covariates included in the linear regression analyses are age, sex, blood cell distributions and technical effects (hybridization batch and plate position).

**Supplementary figure 1.** Correlation blood cell count covariates with principal component 1 to 8

*Green square = CD8+ T cells, Red circle = CD4+ T cells, Blue asterisk = Natural Killer cells, white circle = B cells, black triangle = Granulocytes, Red triangle = Monocytes.*

**Supplementary figure 2.** Correlation covariates with principal component 1 to 8

*Green square = gender, Red circle = age, Blue asterisk = PD, black triangle = hybridization batch, Purple square with cross = Plate position*


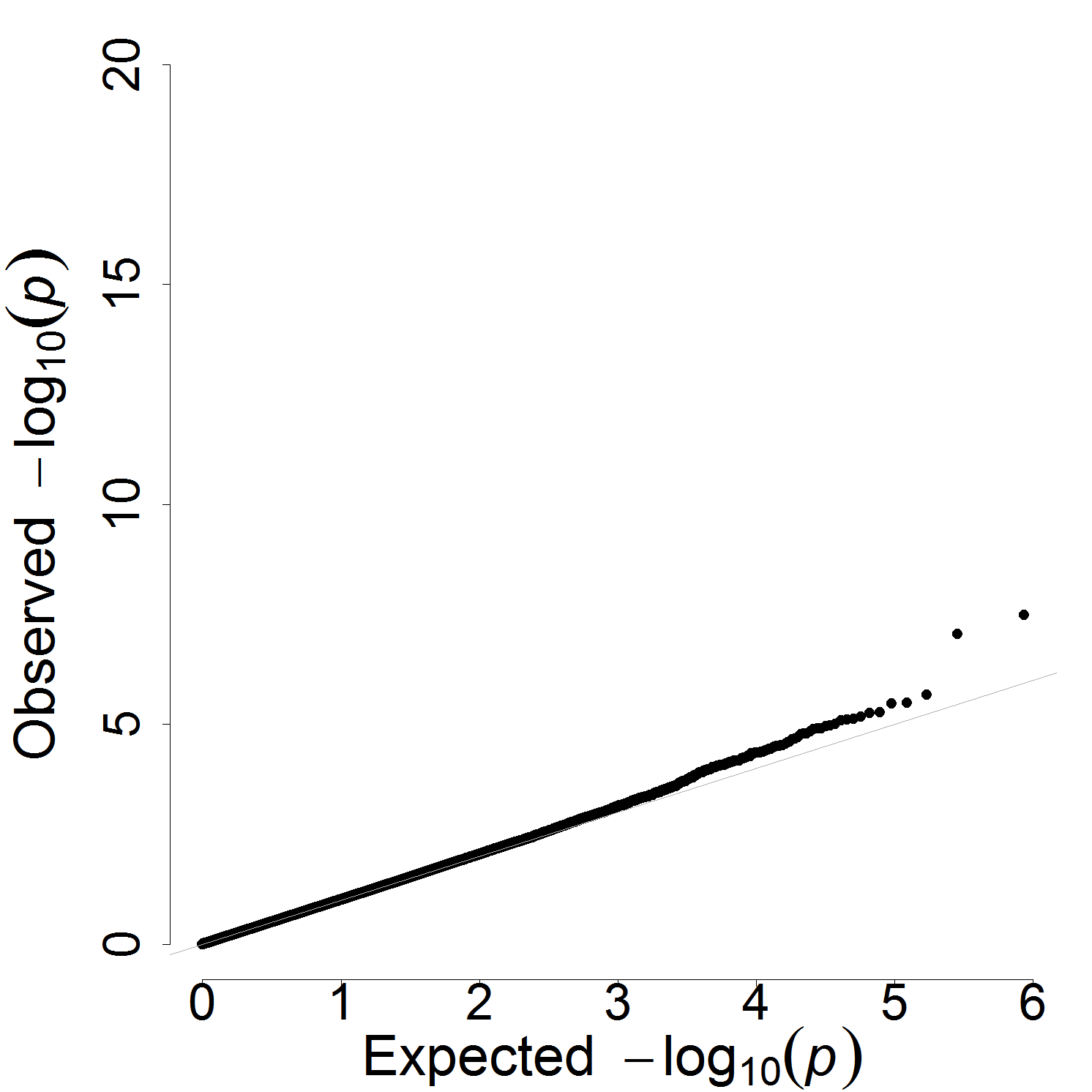


**Supplementary figure 3.** Q-Q plot of EWAS p-values for DMP analyses, corrected with BACON, on the continuous variable of perceived discrimination. Lambda = 1.02

**References**

1 Houseman, E. A. *et al.* DNA methylation arrays as surrogate measures of cell mixture distribution. *BMC bioinformatics* **13**, 1 (2012).
